# Lipopeptide mediated biocontrol activity of endophytic *Bacillus subtilis* against fungal phytopathogens

**DOI:** 10.1186/s12866-019-1440-8

**Published:** 2019-04-02

**Authors:** Dibya Jyoti Hazarika, Gunajit Goswami, Trishnamoni Gautom, Assma Parveen, Pompi Das, Madhumita Barooah, Robin Chandra Boro

**Affiliations:** Department of Agricultural Biotechnology, Assam Agricultural University, 785013, Jorhat, India

**Keywords:** Antifungal activity, *Bacillus subtilis*, Biological control, FAME, LC-MS, Surfactin

## Abstract

**Background:**

The use of chemical fungicides against fungal pathogens adversely affects soil and plant health thereby resulting in overall environmental hazards. Therefore, biological source for obtaining antifungal agents is considered as an environment-friendly alternative for controlling fungal pathogens.

**Results:**

In this study, seven endophytic bacteria were isolated from sugarcane leaves and screened for its antifungal activity against 10 fungal isolates belonging to the genera *Alternaria, Cochliobolus, Curvularia, Fusarium, Neodeightonia, Phomopsis* and *Saccharicola* isolated from diseased leaves of sugarcane. Among the seven bacterial isolates, SCB-1 showed potent antagonistic activity against the tested fungi. Based on the phenotypic data, Fatty Acid Methyl Esters (FAME) and 16S rRNA gene sequence analysis, the isolate SCB-1 was identified as *Bacillus subtilis*. The bacterial isolate was screened negative for chitinase production; however, chloroform and methanol extracts of the bacterial culture caused significant inhibition in the growth of the fungal isolates on semisolid media. Volatile component assay showed highest inhibitory activity against *Saccharicola bicolor* (SC1.4). A PCR based study detected the presence of the genes involved in biosynthesis of surfactin, bacillaene, difficidin, macrolactins and fengycin. Mass spectrometric analysis of the bacterial extract detected the presence of antifungal lipopeptide surfactin, but other metabolites were not detected. The biocontrol activity of the bacterial isolate was established when bacterial pretreated mung bean seeds were able to resist *Fusarium* infection, however, the untreated seeds failed to germinate.

**Conclusion:**

The antifungal potential of isolate *Bacillus subtilis* SCB-1 was established against taxonomically diverse fungal pathogens including the genera *Saccharicola*, *Cochliobolus, Alternaria* and *Fusarium*. The potent antifungal compound surfactin as well as volatiles produced by the bacterial isolate could be responsible for its bio-control activity against fungal infections.

**Electronic supplementary material:**

The online version of this article (10.1186/s12866-019-1440-8) contains supplementary material, which is available to authorized users.

## Background

Plant pathogenic microorganisms are a major threat to the stability of agriculture and ecosystem. The course of human history has been altered due to plant disease epidemics triggered by fungi and the fungal-like oomycetes. In the nineteenth century, Irish potato famine killed nearly 1.5 million people, which had ruined the economy of the English government [1, 2]. In the twentieth century, Dutch elm blight and chestnut blight had affected the urban and forest landscapes [3]. West Bengal of India and Bangladesh is known to suffer the great Bengal famine in 1943 due to the epidemic outbreak of *Helminthosporium oryzae* in rice in the year 1942 [4].

Fungal phytopathogens are challenging to control because of their diverse host spectra, as well as their soilborne nature [5]. Chemical fungicides are commonly used in higher doses to manage the phytopathogens. However, increasing use of chemical fungicides have resulted several undesirable effects, such as development of resistance in pathogens as well as non-targeted environmental impacts of these chemicals. In recent years, large numbers of these synthetic fungicides have been banned in the western world due to undesirable features such as high toxicity [6]. Therefore, alternate measures are essential for long-term and environment-friendly control of the fungal phytopathogens. The use of antagonistic microbes in biological control will not only provide an efficient control of the plant pathogens, but are also harmless to the environment.

Endophytic bacteria serve crucial role in plant health and disease management. Endophytic bacteria occur at lower population densities than pathogens, and generally do not induce any hypersensitive response in their host, indicating that they are not considered by the host plant as pathogens [7]. Several endophytic and rhizospheric bacteria have been reported for their beneficial effects in crop health [8–11]. The bacterial genera that have been reported as endophytes in different plants include *Bacillus*, *Burkholderia*, *Cellulomonas*, *Clavibacter*, *Curtobacterium*, *Paenibacillus*, *Pseudomonas*, *Rhizobium*, *Serratia*, etc. [12–14]. Most endophytes originate from the rhizosphere or phyllosphere; however, vertical transmission through seeds is also reported for some bacterial species [15]. Many endophytic bacteria show antagonistic potential against fungal phytopathogens. Endophytes can also be beneficial by producing a range of natural products that could be harnessed for potential use in medicine, agriculture or industry [16].

In the present study, we characterized the antifungal activity of an endophytic bacterial isolate *Bacillus subtilis* SCB-1 and the major antifungal component produced by the isolate was identified as surfactin.

## Methods

### Isolation of fungal pathogens

Sugarcane leaves and stems were collected from different regions of Golaghat district, Assam. Fungal communities were isolated from infected sugarcane leaves and internodes. After two washes with distilled water and surface sterilization with 0.1% Mercuric Chloride, small sections of the infected parts were inoculated on potato dextrose agar (PDA) plates and incubated for 7 days at 28 °C. Hyphal tips of the fungal colonies were sub-cultured in fresh PDA plates to obtain pure cultures.

### Identification of fungal pathogens

The isolates were identified based on the sequence of the ribosomal Internal Transcribed Spacer (ITS) region of the fungal isolates. The ITS region of the fungal genome was amplified with a pair of universal primers: ITS1: 5′-TCCGTAGGTGAACCTGCGG-3′ and ITS4: 5′-TCCTCCGCTTATTGATATGC-3′ [17]. The PCR thermal profile was as follows: initial denaturation at 94 °C for 3 min; 35 cycles of denaturation step at 94 °C for 30 s, annealing at 50 °C for 30 s, extension at 72 °C for 45 s; and a final extension step at 72 °C for 7 min. The PCR amplicons were purified using GenElute™ PCR Clean-Up Kit (Sigma-Aldrich, USA) and the purified products were sequenced through external vendor (BioServe Biotechnologies, India). The reads obtained after sequencing were assembled and compared with references at GenBank, NCBI using BLAST to obtain their identity. Major pathogens were shortlisted and used as test organisms in the assessment of antifungal activity.

### Isolation of the bacterial endophytes

The bacterial endophytes were isolated form uninfected sugarcane leaves collected from different regions of Golaghat district, Assam. Leaf samples were surface sterilized and crushed in saline water and isolated using serial dilution technique on Tryptic Soy Agar. Plates were incubated for 24 h at 30 °C, and individual colonies with visible differences in colony morphology were sub-cultured to maintain the pure cultures.

### Screening for antifungal activity

Isolated bacterial strains were screened for antifungal activity against the pathogenic fungal isolates using dual culture technique [18]. Mueller Hinton Agar (MHA, HiMedia, India) plates were inoculated with five-day-old culture disks (5 mm diameter) of test fungi at one corner and a bacterial isolate was inoculated at opposite corner. These plates were incubated for 7 days at 28 °C. Antifungal activity expressed in terms of the distance of inhibition was calculated by subtracting the distance (mm) of fungal growth in the direction of an antagonist colony from the antagonist growth radius. The width of inhibition zone between the pathogen and antagonist was ranked as: + for 2–5 mm; ++ for 5–10 mm; +++ for > 10 mm [19]. Amongst the seven isolates tested, only isolate SCB-1 showed antifungal activity against the fungal pathogens. Therefore, the isolate SCB-1 was selected for further studies.

### Characterization of the endophytic bacterial isolate SCB-1

The bacterial isolate SCB-1 showing potent antagonistic activity against the test fungi was selected for characterization. Bacterial isolate was identified based on their colony and cell morphology, biochemical properties and molecular properties. Morphological and biochemical parameters were studied according to the standard protocols [20]. Gas Chromatographic analysis of Fatty Acid Methyl Esters (GC-FAME) was carried out in a 7820A GC System (Agilent Technologies, USA) according to the manufacturer’s protocol. GC-FAME profiling was performed with the MIDI Microbial Identification Sherlock software according to the manufacturer’s instructions.

Molecular identification of the bacterial isolate was carried out based on the sequence of 16S rRNA gene. Genomic DNA was isolated from the bacteria and the 16S rRNA gene was amplified using universal PCR primers: 16S-F (5′-AGAGTTTGATCCTGGCTCAG-3′) and 16S-R (5′-ACGGCTACCTTGTTACGACTT-3′) [21]. The PCR thermal condition used: initial denaturation at 94 °C for 3 min; 35 cycles of denaturation step at 94 °C for 30 s, annealing at 50 °C for 30 s, extension at 72 °C for 1 min 30 s; and a final extension step at 72 °C for 7 min. The amplified PCR product was cloned into pGEM-T Easy Vector System I and sequenced through external vendor (BioServe Biotechnologies, India) using vector derived SP6 and T7 primers. Sequencing reads were assembled and compared with references at GenBank, NCBI using BLAST. A phylogenetic tree was constructed using MEGA 6.0 [22]. The 16S rRNA gene of the bacterial isolate was aligned with the standard reference sequences of different bacterial strains obtained from GenBank. A neighbor-joining tree was built using Kimura-2 parameter model with 1000 bootstrap replications [23]. The sequence was finally submitted to GenBank and accession number obtained.

### Preparation of culture supernatant and metabolite extraction

Liquid culture of the bacterial isolate was prepared by growing in Luria Bertani broth supplemented with 0.5% colloidal chitin [24] for 7 days at 30 °C with shaking at 170 rpm. The culture broth was centrifuged at 6000 rpm for 20 min. The supernatant was then filtered through Whatman filter paper No. 2 and the filtrate was acidified with 1 N HCl to pH 3.0 followed by extraction with an equal volume of chloroform: methanol (1:1). The chloroform soluble organic fraction was separated and concentrated under vacuum and dissolved in methanol or dimethyl sulphoxide (DMSO) according to requirements. The stock solutions were filter-sterilized using 0.22 μm PVDF membrane syringe filter (GE healthcare, USA) and stored at 4 °C until use.

### Antifungal assay

The in vitro antifungal activity of the bacterial crude extract was evaluated by inoculating mycelial plugs (5 mm diameter) taken from the freshly growing fungal cultures onto the center of PDA amended with 0, 250 or 500 ppm of DMSO solubilized chloroform extract. The plates were incubated at 28 °C for 3 to 5 days. The percentage of growth inhibition was calculated using the following formula:

$$ \%\mathrm{Inhibition}=\left[\left(\mathrm{A}-\mathrm{B}\right)/\mathrm{A}\right]\times 100 $$

(Where, A denotes the growth diameter of the fungi in control culture medium and B denotes the growth diameter in culture medium amended with the extracted organic fraction).

Furthermore, the direct inhibitory effect of the crude extract was studied using 24-well culture plates. Fungal strains were pre-cultured in PDB medium at 28 °C for 2 days and 200 μl of each fungal culture was transferred to the respective well of the culture plate. To each well, the extracted organic fraction (dissolved in DMSO) was then added upto final concentrations of 250 or 500 ppm. In the control wells, same volume of DMSO was added. The plates were sealed and incubated at 28 °C for 2 days. Hyphae were stained with lactophenol cotton blue and observed at 100X magnification under oil emersion objective of Olympus BX51 microscope.

### Screening for hydrolytic enzymes

The bacterial strain was screened for production of hydrolytic enzymes. The α-amylase activity was qualitatively determined in starch agar containing 2 g/l soluble starch, 5 g/l peptone, and 15 g/l agar. After 24 h of incubation, plates were flooded with iodine and washed with distilled water. Presence of clear zone surrounding the bacterial colonies was considered as α-amylase positive. For detection of cellulase activity, carboxymethylcellulose (CMC) agar containing 10 g/l CMC, 5 g/l yeast extract, 5 g/l peptone and 15 g/l agar was used. Activity was detected by same method as that of α-amylase. Chitinase activity was detected in King’s B agar base (HiMedia, India) supplemented with 1% colloidal chitin. Protease activity was detected in Skimmed milk agar (HiMedia, India) and presence of clear zone after 48 h of incubation was considered as positive.

### Assay of antifungal volatile components

The antifungal volatile components assay was carried out according to the method described by Yuan et al. (2012) [25] with some modifications. The bacterial strain was grown overnight in LB medium and 100 μl of the culture was spread on nutrient agar (NA) plates. A 5 mm mycelial plug of each fungal strain was cut from the margin of an actively growing culture and placed in the centre of a PDA plate. The PDA plate containing the mycelial plug was inverted over the NA plate with 100 μl bacterial cultures, sealed with Parafilm and incubated at 28 °C. The PDA plate containing mycelial plug of each fungus inverted over an un-inoculated NA plate was used as control. The diameter of fungal colony was measured at an interval of every 24 h, and compared to the control, over a period of 5 days.

### Detection of secondary metabolite biosynthetic genes

*Bacillus subtils* has been reported to produce different secondary metabolites having antifungal activity [26, 27]. Therefore, eight genes viz. *baeR, dfnD, srfA, mlnA, dhbD, bacD, ituA* and *fenA* belonging to the eight different secondary metabolite biosynthetic pathways [28–30] were selected for screening through PCR based method. Reference nucleotide sequences were retrieved from the GenBank, NCBI (http://www.ncbi.nlm.nih.gov/genbank). The PCR primers (Table 1) were designed based on the reference sequences of the respective genes using IDT primerquest tool available at https://www.idtdna.com/PrimerQuest. Genomic DNA from *Bacillus subtilis* SCB-1 was used as template for PCR amplification of the secondary metabolite biosynthetic genes. The PCR reaction was performed using EmeraldAmp® GT PCR Master Mix (Takara, Japan) in 50 μl reaction volume containing 10 pmol of each primer and 50 ng of genomic DNA. The PCR condition was: initial denaturation at 94 °C for 3 min; 35 cycles of denaturation step at 94 °C for 45 s, annealing temperature (Table 1) for 30 s, extension at 72 °C for 1.5 min; and a final extension step at 72 °C for 7 min. The amplified products were analyzed on a 1.2% agarose gel.

**Table 1:** Primer sequences used for the amplification of secondary metabolite biosynthetic genes

Sl No	Primer	Sequence (5*′ –* 3*′*)	Annealing temperature used (°C)	Approximate product Size (base pairs)
1	BaeR-F	AGACTCCACCAAGGCAAATC	55	990
	BaeR-R	CAGCGGCTTCATGTCATACT		
2	DfnD-F	CAGGCGGAATAGGAGAAGTATG	54	900
	DfnD-R	CGGCAGCCGATTGAAATAAC		
3	SrfA-F	GCTGATGATGAGGAGAGCTATG	55	890
	SrfA-R	GATGGTCGATACGTCCGATAAA		
4	MlnA-F	GGCAGGGTCATACCTCATAATC	55	920
	MlnA-R	AGCAGACTTTCGGTCTCATTC		
5	DhbE-F	GCTGGAGGAAGAGTGGTATTATC	54	940
	DhbE-R	CAGTAAATGAAGCGGCGTTATG		
6	BacD-F	CCGGCGTCAAGTCTATCAAA	54	670
	BacD-R	CATGGCTCCTGCTCCAATAA		
7	ItuA-F	CGGGAAACAACAGGCAAATC	55	980
	ItuA-R	CGTCACCAGCGGTGTAAATA		
8	FenA-F	CATTCATCCTGGAGACCCTATTC	55	960
	FenA-R	TAAGACCGCAGGCATGTTATAG		

### Identification of the bioactive components

The extract from the bacterial cell free supernatant was dissolved in methanol and filtered through 0.22 μm PVDF membrane syringe filter (GE healthcare, USA). The major components of the extracts were separated using an Acquity Waters UPLC system installed with a BEH C18 column. A 10 μl aliquot of the sample was injected into the column and the column was eluted at a flow rate of 1.0 ml/min with methanol: water (20: 80) at 0 min and gradually increasing the polarity to methanol: water (80: 20) at 15 min which was further retained till 20 min. The fractions were detected by using an ESI-QTOF-Mass Spectrometer connected to the UPLC system (Waters Corporation, USA).

### Determination of biocontrol potential of SCB-1

Mung bean (*Vigna radiata* L.) seeds were used to study the biocontrol potential of SCB-1 during germination. Seeds were surface sterilized with 1% sodium hypochlorite solution and soaked overnight in the bacterial suspension. The bacterial suspension was prepared in 0.85% sodium chloride solution adjusted to 0.6 OD_600_ prior to application. Untreated seeds were soaked overnight in 0.85% sodium chloride solution. Spore suspension from three fungal strains of *Fusarium* viz. *F. oxysporum* SC7.1, *F. verticillioides* SC8.1 *and Fusarium* sp. SC9.1 were prepared with initial concentration of ~ 10^4^ spores/ml. Bacteria treated and untreated seeds were soaked in the respective spore suspensions for 30 min. Bacteria treated and untreated seeds without fungal inoculum were used as positive controls. The germination percentage, root and shoot lengths were measured after 48 h of fungal inoculation.

### Statistical analysis

The data obtained from antifungal assay of bacterial extract was analyzed using student’s *t*-test in Microsoft Excel and *p* ≤ 0.05 was considered as significant. Data for antagonistic activity by volatile organic components and seed germination assay were analyzed in SPSS 20.0 software by one-way Analysis of Variance (ANOVA). Duncan multiple range test was performed to study the level of significance among the treatments (*p* ≤ 0.05).

## Results

### Identification of the fungal pathogens

A total of 24 fungal isolates were obtained from the infected portions of sugarcane leaves and stems. These isolates were identified based on similarities of their ITS sequences, as revealed by BLAST analysis. The list of identified fungal isolates is shown in Additional file 1: Table S1. Based on the earlier reports, ten of these fungal isolates were marked as sugarcane pathogens and therefore were used as test fungi for further study.

### Antagonistic activity of the bacterial isolate against fungal isolates

**Fig. 1:**
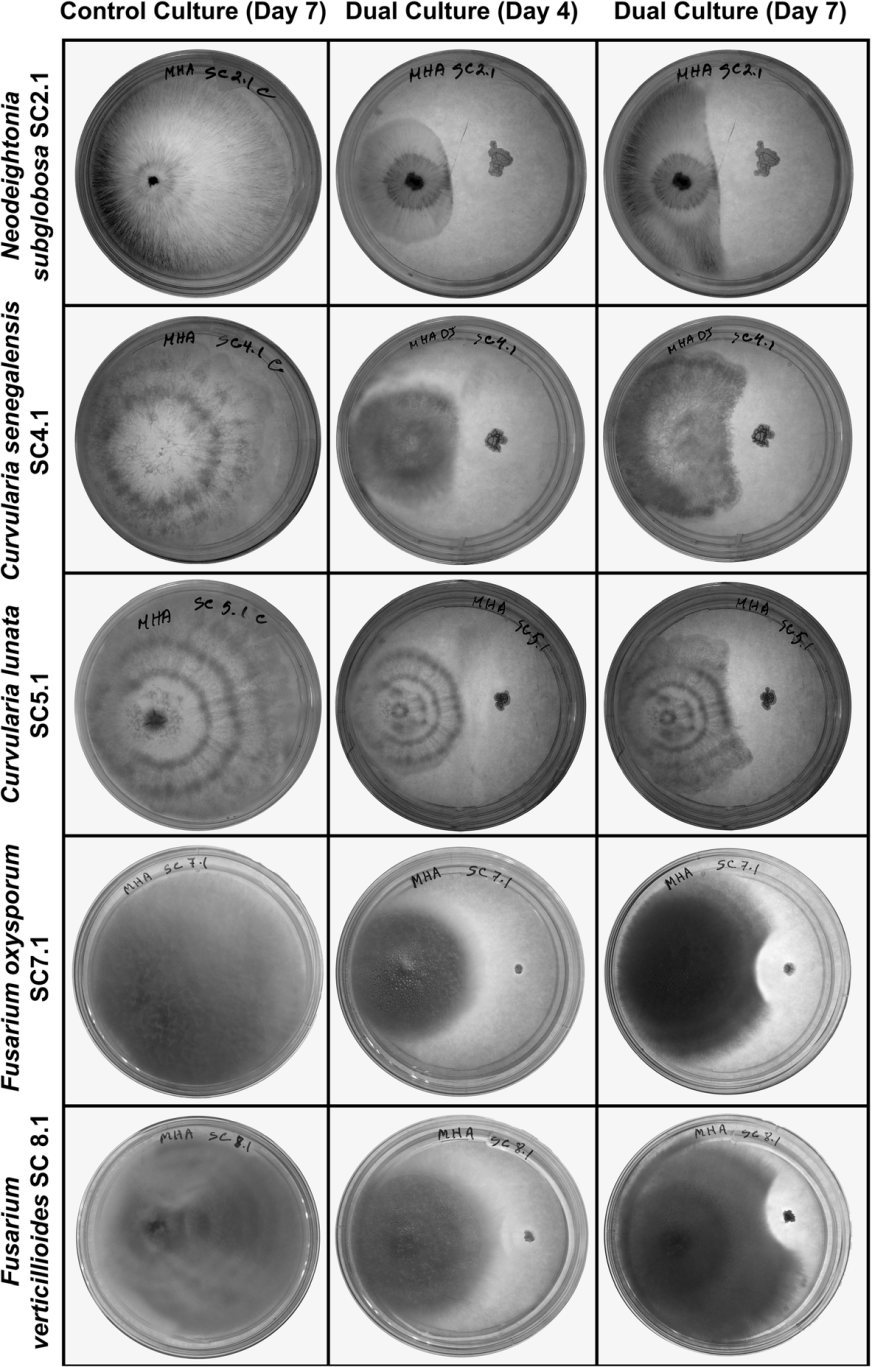
Antagonistic activity of isolate SCB-1 against different sugarcane phytopathogens. The dual culture represents inoculation of SCB-1 (on the right side of each dual culture plate) with the respective fungal strains

Seven isolates of endophytic bacteria were obtained from healthy sugarcane leaves. All the isolated bacteria were used to study their antagonistic potential against the test fungi. Out of the seven bacterial isolates, only one isolate (SCB-1) showed antagonistic effect on all the tested fungal isolates (Table 2; Fig. 1a). The zone of inhibition persisted up to 14 days of inoculation, and higher inhibition distance (15.67 ± 0.58 mm) was recorded against SC5.1 (*Curvularia lunata*) and lowest inhibition distance (9.00 ± 1.00 mm) was recorded against SC8.1 (*Fusarium verticillioides*).

**Table 2:** Antagonistic activity of isolate SCB-1 against tested phytopathogens

Sample Code	Organism’s name	Distance of inhibition (mm)	Antagonistic Activity
SC1.4	*Saccharicola bicolor* SC1.4	13.33 ± 0.58	+++
SC2.1	*Neodeightonia subglobosa* SC2.1	15.00 ± 1.00	+++
SC2.3	*Cochliobolus hawaiiensis* SC2.3	11.67 ± 0.15	+++
SC4.1	*Curvularia senegalensis* SC4.1	12.67 ± 1.53	+++
SC4.2	*Phomopsis* sp*.* SC4.2	13.67 ± 0.58	+++
SC5.1	*Curvularia lunata* SC5.1	15.67 ± 0.58	+++
SC6.2	*Alternaria alternata* SC6.2	15.33 ± 1.15	+++
SC7.1	*Fusarium oxysporum* SC7.1	9.67 ± 1.15	++
SC8.1	*Fusarium verticillioides* SC8.1	9.00 ± 1.00	++
SC9.1	*Fusarium* sp. SC9.1	10.00 ± 1.00	++

### Identification of the bacterial strain with antifungal activity

Since SCB-1 showed the highest zone of inhibition when tested against the fungal pathogens, it was selected for identification and further study. The preliminary observations revealed that, the bacterial isolate was Gram positive, rod shaped, motile when grown in nutrient agar (NA) medium. Colonies were opaque, whitish in colour, and glisterning when grown in PDA and chitin agar medium. The detailed results of biochemical tests are given in the Additional file 1: Table S2. In addition, based on the result of FAME and 16S ribosomal RNA gene sequence analysis, the isolate SCB-1 was identified as *Bacillus subtilis*. The 16S rRNA gene sequence was submitted to NCBI (accession number MF893335.1). A phylogenetic tree was constructed using Neighbour-Joining method, which suggested the close relationship of the isolate SCB-1 with its reference *B. subtilis* strain (Additional file 1: Figure S1). The tree also indicated that the isolate SCB-1 is also closely related to *B. amylolequifeciens*.

### Antifungal activity of the chloroform and methanol extracts of SCB-1

**Fig. 2:**
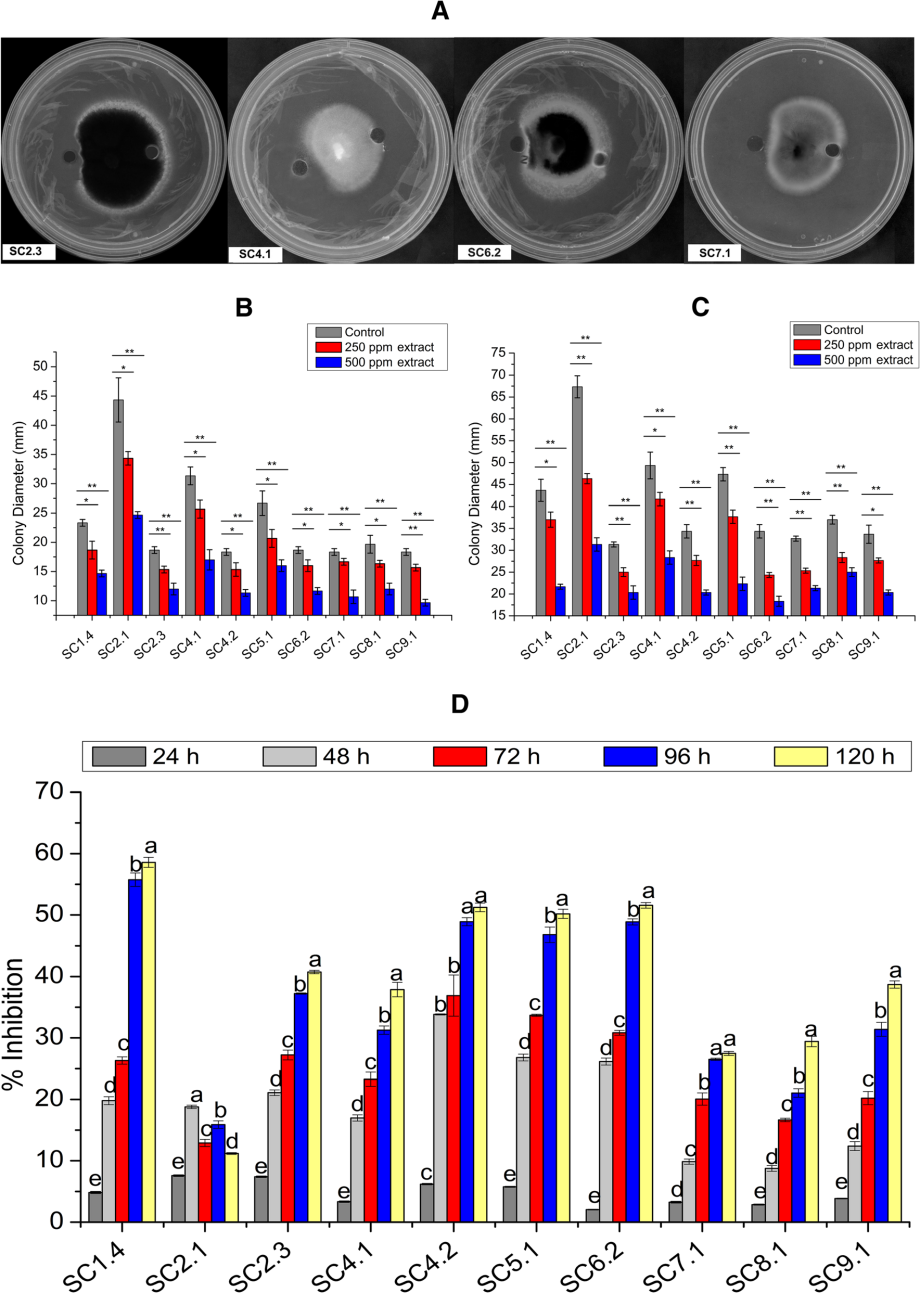
Antagonistic activity of the SCB-1 culture supernatant, crude extracts, and volatile organic components. **a** Antifungal activity of the culture supernatant against four test fungi (*Cochliobolus hawaiiensis* SC2.3, *Curvularia senegalensis* SC4.1, *Alternaria alternata* SC6.2 and *Fusarium oxysporum* SC7.1) on PDA plates. Photographs were taken after 72 h of inoculation. **b** and **c** Growth diameter of the fungal colonies challenged with 0 ppm (control), 250 ppm and 500 ppm of bacterial crude extract after 48 h and 72 h, respectively. Asterisk (*) and double Asterisk (**) indicates level of significance over control with ***p*** ≤ **0.05** and ***p*** ≤ **0.01**, respectively (*t*-test, Microsoft Excel 2013). **d** Inhibitory activity of bacterial volatile organic components against the test fungi. Results are represented as the inhibition percentage of the colony diameter of the test fungi at different time interval. The different letters (a-e) above the bars represent significant differences (***p*** **≤ 0.05**) in the inhibition percentage for individual test fungi, otherwise non-significant

The bacterial supernatant also showed significant antifungal activity. The hyphal growth was inhibited towards the wells loaded with bacterial supernatant, whereas, control wells were covered by fungal hyphae (Fig. 2a). The chloroform extracted organic fractions showed strong inhibition against all the selected fungal strains. Inhibition was found to be proportional to the concentration of the chloroform extract used in all the cases (Fig. 2b-c). Microscopic observation of the fungal hyphae revealed the alteration of hyphal morphology in all the fungal strains. The normal growth and branching patterns of the hyphae were disrupted with the increasing concentrations of chloroform extracts showing abnormal bending, higher septation and vegetative spore formation in some cases. In contrast, normal hyphal growth with normal septation was observed in the controls.

### Volatile component inhibition assay

Antifungal volatile compounds were produced by the bacterial strain which could inhibit the growth of fungal colonies exposed over the bacterial cultures. The volatiles were able to reduce the colony diameters of nine fungal strains except SC2.1. The results are represented in Fig. 2d as the percent inhibition of the colonies in the bacteria treated plates compared to the control plates, which indicated significant growth inhibition from 48 h onwards for those nine fungal pathogens (*p*-value < 0.05). However, in SC2.1 culture plates, hyphal density of the colonies was lower than the control plates.

### Lytic enzyme assay of the bacterial culture supernatant

Upon screening of hydrolytic enzymes, the bacterial isolate was found to have α-amylase, cellulase and protease activity, while chitinase activity was not detected. Furthermore, hydrolytic enzyme assay using spectrophotometric method indicated the significant production of α-amylase, cellulase and protease.

### Detection of antifungal biosynthetic genes

**Fig. 3:**
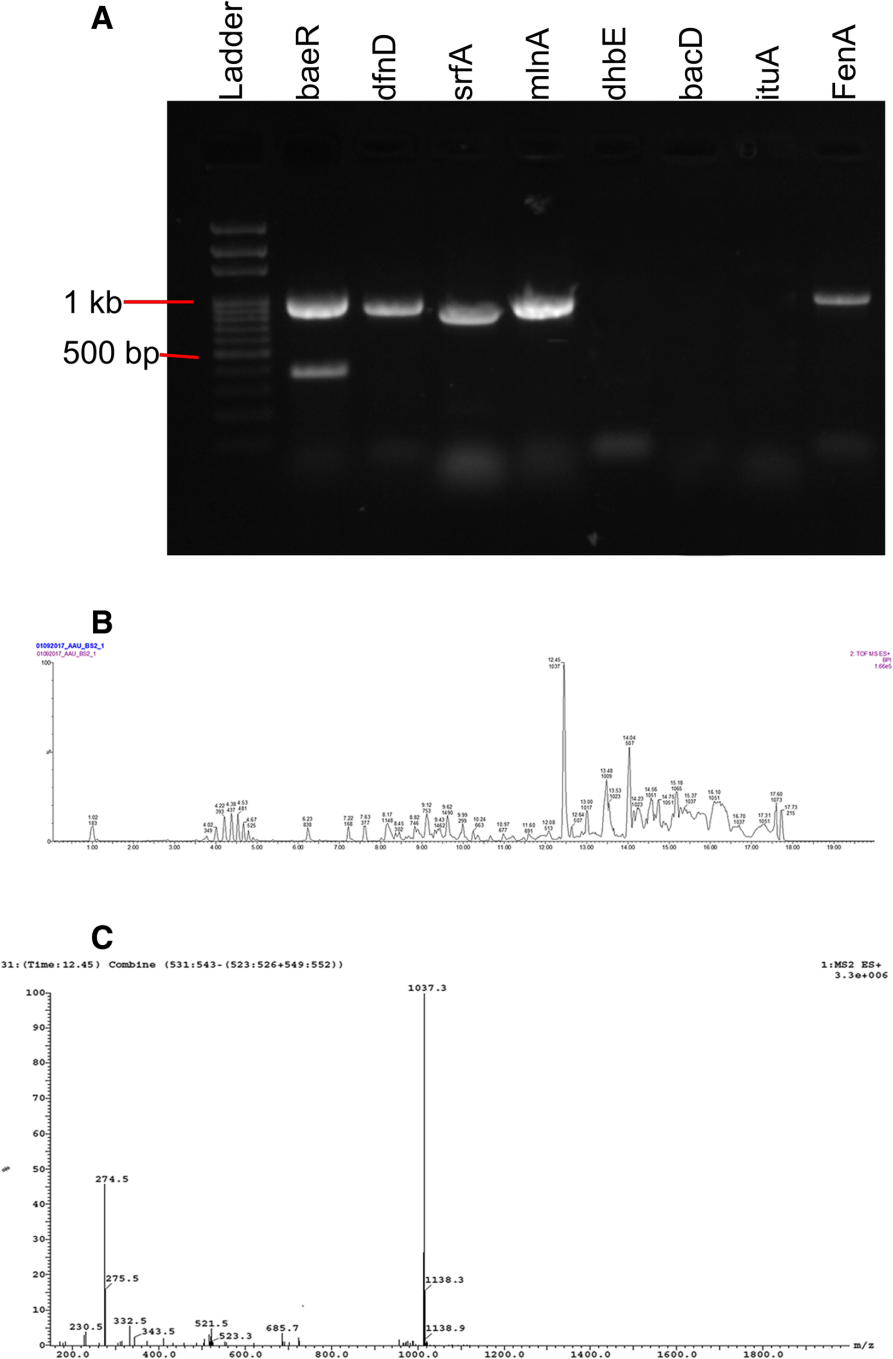
Detection of secondary metabolites produced by bacterial isolate SCB-1. **a** PCR amplified products of the eight secondary metabolite biosynthetic genes on 1.2% agarose gel. **b** UPLC chromatogram of the bacterial crude extract. **c** Mass spectra of surfactin corresponding fraction eluted at retention time of 12.45 min

Secondary metabolite biosynthetic genes in the genome of SCB-1 were investigated using PCR. The results suggested the presence of surfactin biosynthetic gene *srfA* along with four other genes namely *baeR* (gene for bacillaene biosynthesis), *dfnD* (gene for difficidin biosynthesis), *mlnA* (gene involved in biosynthesis of macrolactins), and *fenA* (gene for fengycin biosynthesis). The amplified products of band size ~ 990 bp for *baeR* gene, ~ 900 bp for *dfnD* gene, ~ 890 bp for *srfA* gene, ~ 920 bp for *mlnA* gene, and ~ 960 bp for *fenA* gene. However, the expected amplicon size for *dhbE* gene (~ 940 bp), *bacD* gene (~ 670 bp) and *ituA* gene (~ 980 bp) were not detected (Fig. 3a).

### Analysis of the antifungal compounds

The LC-ESI-MS analysis of the bacterial extract indicated the presence of an antifungal compound surfactin. The major fraction in the extract had a retention time of 12.45 min. The UPLC total ion chromatogram of the bacterial extract is shown in Fig. 3b. The mass spectral data revealed the presence of surfactin C-15 (molecular mass: 1036.3) with an m/z of 1037.3 (M + H)^+^ (Fig. 3c). Besides surfactin C-15, low abundance of surfactin C-13 and surfactin C-14 were also recorded. On the other hand, no significant peak for bacillaene, difficidin, macrolactins or fengycin was detected in the LC-ESI-MS analysis.

### Biocontrol potential of SCB-1 in germinating seeds

**Fig. 4:**
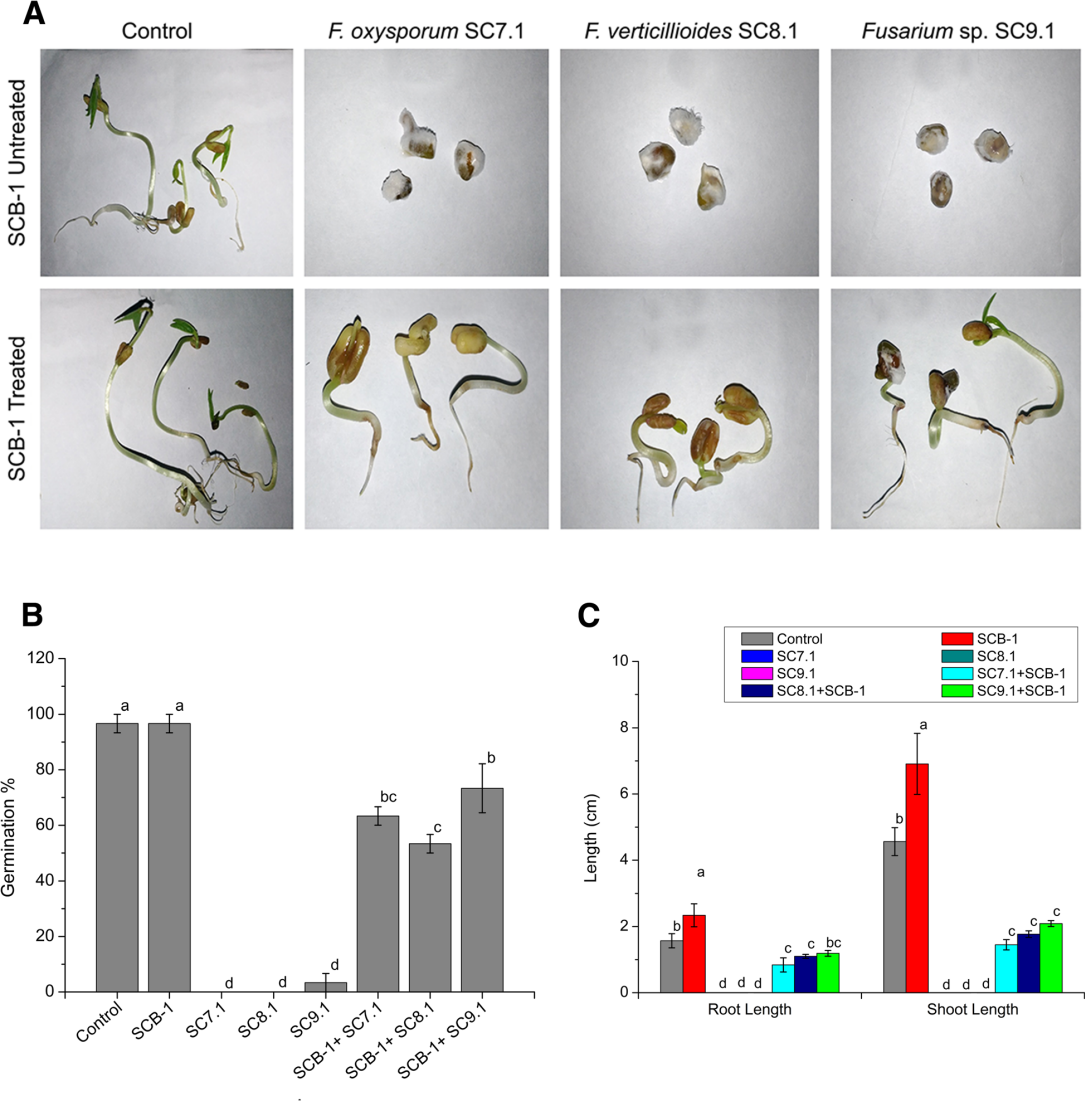
Biocontrol potential of SCB-1 against three Fusarium isolates on germinating seeds mung bean. **a** Photographs of bacteria untreated and treated germinating seeds after 48 h of fungal inoculation. **b** Graph representing germination percentage of mung bean seeds after different treatments. **c** Graph representing the root and shoot lengths of germinating seeds after different treatments. The different letters (**a**-**d**) above the bar represent significant difference (***p*** ≤ **0.05)** in the germination percentage, root lengths and shoot lengths of germinating seeds, otherwise non-significant

The bacterial isolate was able to inhibit the fungal infection in the germinating mung bean seeds. The seeds that were treated with spore suspension of the three test fungi alone failed to germinate. However, the bacteria pre-treated seeds showed resistant towards the test fungi as evident from root development (Fig. 4a). The germination percentage for each set is represented in Fig. 4b. The average root and shoot lengths of the germinating seeds are shown in Fig. 4c.

## Discussion

Endophytic bacteria are not new to the microbiologists and have been reported from vast number of vascular plants [9, 31]. A single plant can hurbour several endophytic bacteria or fungi, among which at least one species show host specificity [31]. Although, endophytic bacteria enhance the fitness of the host plant, some of these bacterial species also contribute to the resistance of their host against pathogens. We were interested in the bacterial endophytes that can minimize fungal infections and can be used as biocontrol agents against phytopathogenic fungi.

For this study, we selected sugarcane plants for isolation of fungal pathogens from the leaves and stems. Sugarcane (*Saccharum officinarum* L.) is being of the most important commercial crops in India contributes nearly 70% for sugar production and provides the base material crucial for many other industries. This crop is attacked by several pathogens and some of which cause severe yield losses leading to negative impact on sugarcane production, as well as in the sugar industry. There are about 56 diseases of sugarcane which have been reported and out of these 40 are caused by fungi, several of which can cause economic loss [32]. We isolated 21 fungi from the leaves and 3 fungi from the internodes of sugarcane. The fungal community in sugarcane observed in our study includes some important leaf pathogens that has been previously reported [33–35].

Bacteria that colonize the plant tissues without causing apparent harm to the plant can be isolated from surface-sterilized plant tissues, and such bacteria could be considered endophytes [7]. In our study, seven bacterial foliar endophytes were isolated from healthy leaves of sugarcane plants. Out of these seven bacterial isolates, only one isolate (SCB-1) showed antifungal activity against all the tested fungal pathogens. Therefore, this strain was further characterized to divulge its identity. The bacterial isolate was identified as *Bacillus subtilis* based on the morphological and molecular data. Phylogenetic analysis of the 16S rRNA gene also indicated the close relationship of isolate SCB-1 with *B. subtilis* reference strains. Several strains of *Bacillus subtilis* and some other species of the genus are often found as colonizers of the internal tissues of plants [36–39]. It is one of the most important bacterial species known to improve plant growth and development [40, 41]. Different *B. subtilis* strains have been previously used for control of plant diseases including take-all in wheat [42], dumping-off of tomato [43], chestnut blight [10], *Fusarium* root infection [44] and many others.

The culture supernatant was able to inhibit the fungal growth in PDA plates. The antifungal activity exhibited by the isolate SCB-1 was due to the extracellular components secreted into the culture supernatant. Most bacteria produce extracellular bioactive components in the form of enzymes, peptides or metabolites. Many bacteria produce chitinases that target the fungal cell wall and thus show antifungal properties [45–47]. In our case, the isolate SCB-1 showed no chitinase activity in the chitin agar medium. On the other hand, the chloroform and methanol extracts of the culture supernatant showed significant inhibition of the fungal growth. These results suggested that the antifungal activity of the bacteria SCB-1 is due to production of some antifungal compounds, but not through the production of chitinases. Volatile organic component assay indicated the production of volatiles that could inhibit the growth of the tested fungi. Earlier studies also reported that *Bacillus subtilis* and few other members of the genera are known to produce volatile components [25, 48].

We also screened for the presence of secondary metabolite biosynthetic genes in the bacterial genome using gene specific primers. The selected genes belong to different operons of the genomes of various *Bacillus* strains that are involved in the biosynthesis of polyketides and lipopeptides [49]. Presence of non-ribosomal polyketide and lipopeptide biosynthetic genes can be correlated with the biocontrol potential of bacteria [50]. Among the eight tested genes, five genes viz. *baeR*, *dfnD*, mlnA, *srfA*, and *fenA* were detected in the bacterial genome. The former three genes were reported to be involved in the synthesis of bioactive polyketides bacillaene, difficidin and macrolactins and respectively [29, 51]. Bacillaene has been reported to show antagonistic activity against bacteria and fungi [52, 53]. Difficidin and macrolactins are broad spectrum antibiotics produced by *Bacillus* sp. (Schneider et al., [29]). Products of *srfA* and *fenA* are involved in the biosynthetic pathways of surfactin and fengycin, respectively [54, 55] which are well known for their antifungal properties.

Since the PCR based study detected the presence of these five secondary metabolite genes, we wanted to detect the active antifungal compound(s) using LC-ESI-MS. Characterization of the bacterial extract by LC-ESI-MS revealed the presence of surfactin, a lipopeptide biosurfactant known to be produced by many strains of *Bacillus* [56]. This compound belongs to the group of cyclic lipopeptides comprising beta-hydroxyl fatty acids and D−/L- amino acid residues [57, 58]. Surfactin shows superior surface activity and biosurfactant properties. Among the three well known isoforms (C-13 surfactin, C-14 surfactin and C-15 surfactin), the C-15 surfactin (M.W. 1036.3) has been reported to show highest surface activity [57]. The antifungal activity of surfactin is due to its interaction with the cell membrane and disruption of membrane stability [59–62].

Various other non-ribosomally synthesized lipopeptides such as iturins, fengycin etc. shows structural similarities to surfactin [49]. The notable differences among these lipopeptides include the type of the amino acids, their peptide sequences, the branching of the fatty acid chain [63] and their biosynthetic genes present in different operons. The presence of *fenA* and other four polyketide genes have been detected in the bacterial genome, however, fengycin, bacillaene, difficidin, macrolactins and bacillomycin were not detected by LC-ESI-MS. Synthesis of these compounds may be condition specific and therefore were not synthesized during the antagonistic activity against the fungal pathogens. In many cases, surfactin synergistically works with fengycin and other compounds to show antifungal activity against several fungal pathogens [64–66]. We could detect surfactin as the active component, which may solely be responsible for the antifungal activity of the bacterial isolate under study. According to Li et al. (2014) [67] the production of iturin could be affected by the nature of different pathogens, suggesting that *Bacillus* species can use different antibiotics depending on the pathogen encountered. In another study, the lipopeptide profile of three *B. subtilis* strains (AS43.3, AS43.4, and OH131.1) was studied which suggested that surfactin was produced by all three strains at all growth stages, while iturin and fengycin were not produced by strain OH131.1 at any growth stages. Other two strains produced these two compounds only during the stationary growth phase, suggesting that synthesis of these compounds is strain and growth stage specific [68].

*Bacillus subtilis* and few other members of the genera has been used as biocontrol agents against various fungal pathogens in different crop plants [69]. *Fusarium* has been reported to cause root rot and wilt of mung bean and also detected in mung bean as seedborne pathogen [70]. As *Bacillus subtilis* SCB-1 showed remarkable in-vitro antagonistic effect against three *Fusarium* strains, we further tested its efficacy against these strains on germinating mung bean seeds. The ability of SCB-1 pre-treated mung bean seeds to germinate after *Fusarium* inoculation suggested that our bacterial isolate SCB-1 has potent biocontrol activity. Production of surfactin and other volatiles may have important role in the suppression of *Fusarium* infestation on germinating seeds. Surfactin and other lipopeptides produced by *Bacillus subtilis* was reported to play key role against major fungal diseases like damping-off disease caused by *Rhizoctonia solani* and powdery mildew caused by *Podosphaera fusca* [71, 72]. Surfactin A purified from *Bacillus* strains had strong antifungal activity against *Fusarium oxysporum*, *F. moniliforme*, *F. solani*, *Trichoderma atroviride* and *T. reesei* [73].

## Conclusion

Our results established the antifungal potential of the endophytic isolate *Bacillus subtilis* SCB-1. This isolate was found to have antagonistic activity against taxonomically diverse fungal pathogens including the genera *Saccharicola*, *Cochliobolus, Alternaria* and *Fusarium*. The antifungal compounds such as surfactin, as well as volatiles produced by this bacterial strain can be used for control of fungal infections. Furthermore, biocontrol potential of this bacterial isolate against different strains of *Fusarium* has opened up chances for applicability of this strain as promising biocontrol agent in near future.

## Additional file

Additional file 1: **Table S1:** Isolated fungal samples with their identity based on the ITS sequence similarity. **Table S2:** Morphological and Biochemical Characteristics. **Figure S1:** Phylogenetic relationship of *Bacillus subtilis* SCB-1 with their related bacterial strains based on the 16S rRNA gene sequence. The tree was constructed using Neighbour-Joining method incorporated in MEGA6 with 1000 bootstrap replications. **Figure S2:** Hydrolytic enzyme activity of SCB-1 on solid medium. **A.** Amylase activity on starch agar medium. **B.** Cellulase activity on CMC agar medium. **C.** Protease activity on skimmed milk agar medium. **Figure S3:** Enzyme activity against the incubation time of *Bacillus subtilis* SCB-1 in respective culture medium. Error bars represent the standard deviation from three replicates. (DOCX 1100 kb)

## Abbreviations

BLAST: Basic local alignment search tool
bp: Base pair(s)
ESI-QTOF: Electrospray ionization-quad-time of flight
g/l: Gram(s) per liter
h: Hour(s)
HCl: Hydrochloric acid
min: Minute(s)
mm: Millimeter(s)
N: Normal
NCBI: National centre for biotechnology information
ng: Nanogram(s)
OD_600_: Optical density at 600 nm
PCR: Polymerase chain reaction
pmol: Pico mole(s)
PVDF: Polyvinylidene difluoride
rRNA: Ribosomal ribonucleic acid
s: Second(s)
sp.: Species
UPLC: Ultra performance liquid chromatography
μl: Microliter
μm: Micrometer
