# Supplementary material for: Lipopeptide mediated biocontrol activity of endophytic Bacillus subtilis against fungal phytopathogens
Source: BMC Microbiol. 2019 Apr 2;19:71. doi: 10.1186/s12866-019-1440-8 (PMC6444643; doi:10.1186/s12866-019-1440-8)
Supplement: Supplementary file 1 — Table S1: Isolated fungal samples with their identity based on the ITS sequence similarity. Table S2: Morphological and Biochemical Characteristics. Figure S1: Phylogenetic relationship of Bacillus subtilis SCB-1 with their related bacterial strains based on the 16S rRNA gene sequence. The tree was constructed using Neighbour-Joining method incorporated in MEGA6 with 1000 bootstrap replications. Figure S2: Hydrolytic enzyme activity of SCB-1 on solid medium. A. Amylase activity on starch agar medium. B. Cellulase activity on CMC agar medium. C. Protease activity on skimmed milk agar medium. Figure S3: Enzyme activity against the incubation time of Bacillus subtilis SCB-1 in respective culture medium. Error bars represent the standard deviation from three replicates. (DOCX 1100 kb) [file 12866_2019_1440_MOESM1_ESM.docx]

**Journal Name:** BMC Microbiology

**Manuscript Title:** Lipopeptide mediated biocontrol activity of endophytic *Bacillus subtilis* against fungal phytopathogens

**Authors:** Dibya Jyoti Hazarika, Gunajit Goswami, Trishnamoni Gautom, Assma Parveen, Pompi Das, Madhumita Barooah, Robin Chandra Boro*

**Affiliation:** Department of Agricultural Biotechnology, Assam Agricultural University, Jorhat-785013, India

*Corresponding Author’s email: robinboro@gmail.com

**Supporting information**

**Table S1:** Isolated fungal samples with their identity based on the ITS sequence similarity.

| Sl No | Sample code | Isolation source | Identified Name | GenBank Accession No. |
| --- | --- | --- | --- | --- |
| 1 | SC1.1 | Sugarcane leaf | *Trichoderma* sp. SC1.1 | MH087097 |
| 2 | SC1.2 | Sugarcane leaf | *Pennicillium* sp. SC1.2 | MH087098 |
| 3 | SC1.3 | Sugarcane leaf | *Aspergillus niger*. SC1.3 | MH087099 |
| 4 | SC1.4* | Sugarcane leaf | *Saccharicola bicolor* SC1.4 | MH087100 |
| 5 | SC2.1* | Sugarcane leaf | *Neodeightonia subglobosa* SC2.1 | MH087101 |
| 6 | SC2.2 | Sugarcane leaf | *Aspergillus niger* SC2.2 | MH087102 |
| 7 | SC2.3* | Sugarcane leaf | *Cochliobolus hawaiiensis* SC2.3 | MH087103 |
| 8 | SC3.1 | Sugarcane leaf | *Diaporthe phaseolorum ­* SC3.2 | MH087104 |
| 9 | SC3.2 | Sugarcane leaf | *Saccharomyces cerevisiae* SC3.2 | MH087105 |
| 10 | SC3.3 | Sugarcane leaf | *Daldinia eschscholzii* SC3.3 | MH087106 |
| 11 | SC4.1* | Sugarcane leaf | *Curvularia senegalensis* SC4.1 | MH087107 |
| 12 | SC4.2* | Sugarcane leaf | *Phomopsis* sp. SC4.2 | MH087108 |
| 13 | SC5.1* | Sugarcane leaf | *Curvularia lunata* SC5.1 | MH087109 |
| 14 | SC5.2 | Sugarcane leaf | *Curvularia senegalensis* SC5.2 | MH087110 |
| 15 | SC6.1 | Sugarcane leaf | *Diaporthe* sp. SC6.1 | MH087111 |
| 16 | SC6.2* | Sugarcane leaf | *Alternaria alternata* SC6.2 | MH087112 |
| 17 | SC6.3 | Sugarcane leaf | *Trichoderma* sp. SC6.3 | MH087113 |
| 18 | SC6.4 | Sugarcane leaf | *Daldinia eschscholzii* SC6.4 | MH087114 |
| 19 | SC7.1* | Sugarcane stem | *Fusarium oxysporum* SC7.1 | MH087115 |
| 20 | SC7.2 | Sugarcane stem | *Saccharomyces cerevisiae* SC7.2 | MH087116 |
| 21 | SC8.1* | Sugarcane stem | *Fusarium verticillioides* SC8.1 | MH087117 |
| 22 | SC8.2 | Sugarcane stem | *Daldinia* sp. SC8.2 | MH087118 |
| 23 | SC8.3 | Sugarcane stem | *Saccharomyces cerevisiae* SC8.3 | MH087119 |
| 24 | SC9.1* | Sugarcane stem | *Fusarium* sp. SC9.1 | MH087120 |

**Table S2: Morphological and Biochemical Characteristics.**

| **Morphological Characters** | | | | | | | | | | |
| --- | --- | --- | --- | --- | --- | --- | --- | --- | --- | --- |
| **Characters** | | | | **Result** | | | | | | |
| Colony morphology | | | | Dull white, rough, opaque, irregular | | | | | | |
| Gram’s reaction | | | | Gram positive | | | | | | |
| Cell shape, size | | | | Rod shaped, 2-3 µm | | | | | | |
| Sporulation | | | | Positive | | | | | | |
| Biofilm formation | | | | Positive | | | | | | |
|  | | | | | | | | | | |
| **Biochemical Tests using API 50 CHB** | | | | | | | | **Biochemical Tests using API 20** | | |
| **Tests** | | **Result** | **Tests** | | | **Result** | | **Tests** | | **Result** |
| Glycerol | | P | Salicin | | | P | | Catalase | | P |
| Erythritol | | N | Cellobiose | | | P | | ONPG | | P |
| D-Arabinose | | N | Maltose | | | P | | ADH | | N |
| L-Arabinose | | P | Lactose | | | W | | LDC | | N |
| Ribose | | P | Melibiose | | | W | | ODC | | N |
| D-Xylose | | W | Saccharose | | | P | | Citrate | | P |
| L-Xylose | | N | Trehalose | | | P | | H2S | | N |
| Adonitol | | N | Inulin | | | P | | Urease | | N |
| Β-Methyl-D-xyloside | | P | Melezitose | | | N | | TDA | | N |
| Galactose | | P | D-Raffinose | | | W | | Indole | | N |
| D-Glucose | | P | Amidon | | | W | | MR | | N |
| D-Fructose | | P | Glycogen | | | P | | VP | | P |
| D-Mannose | | N | Xylitol | | | N | | Gelatin | | P |
| L-Sorbose | | N | β-Gentiobiose | | | P | | Nitrate | | P |
| Rhamnose | | N | D-Turanose | | | W | | Lechithinase | | P |
| Dulcitol | | N | D-Lyxose | | | N | | Protease | | P |
| Inositol | | W | D-Tagatose | | | N | |  | | |
| Mannitol | | P | D-Fucose | | | N | | **Growth at (°C)** | | |
| Sorbitol | | P | L-Fucose | | | N | | 4 °C | | N |
| Methyl- α -D-mannopyranoside | | N | D-Arabitol | | | N | | 40 °C | | P |
| Methyl-α-D-glucopyranoside | | P | L-Arabitol | | | N | | 50 °C | | N |
| N-Acetylglucosamine | | W | Gluconate | | | N | |  | | |
| Amygdalin | | P | 2-Ketogluconate | | | N | | **Growth on NaCl (%)** | | |
| Arbutin | | P | 5-Ketogluconate | | | N | | 5 % | | P |
| Esculin | | P | **[P= Positive; N = Negative; W= Weak Positive]** | | | | | 7 % | | P |
|  |  |  |  |  |  |  |  | 10 % | | N |
|  | | | | | | | | | | |
| **FAME Analysis Results** | | | | | | | | | | |
| Major fatty acids | 14:00 (1.3), 14:0 iso (3.56), 15:0 iso (32.1), 15:0 anteiso (37.64), 16:1 w7c alcohol (1.06), 16:1 w11c (2.57), 16:00 (3.27), 16:0 iso (2.12), 17:0 iso (4.07), 17:0 anteiso (5.52), 18:00 (1.01), Sum In Feature 4(17:1 iso I/anteiso B) (1.17) | | | | | | Identification | | *Bacillus subtilis* | |
|  |  |  |  |  |  |  | Similarity Index | | 0.607 | |
|  |  |  |  |  |  |  |  | | | |
|  | | | | | | | | | | |
| **16S rRNA Gene Analysis** | | | | | | | | | | |
| BLAST similarity | | | | | Bacillus subtilis | | | | | |
| Identity (%) | | | | | 99 | | | | | |


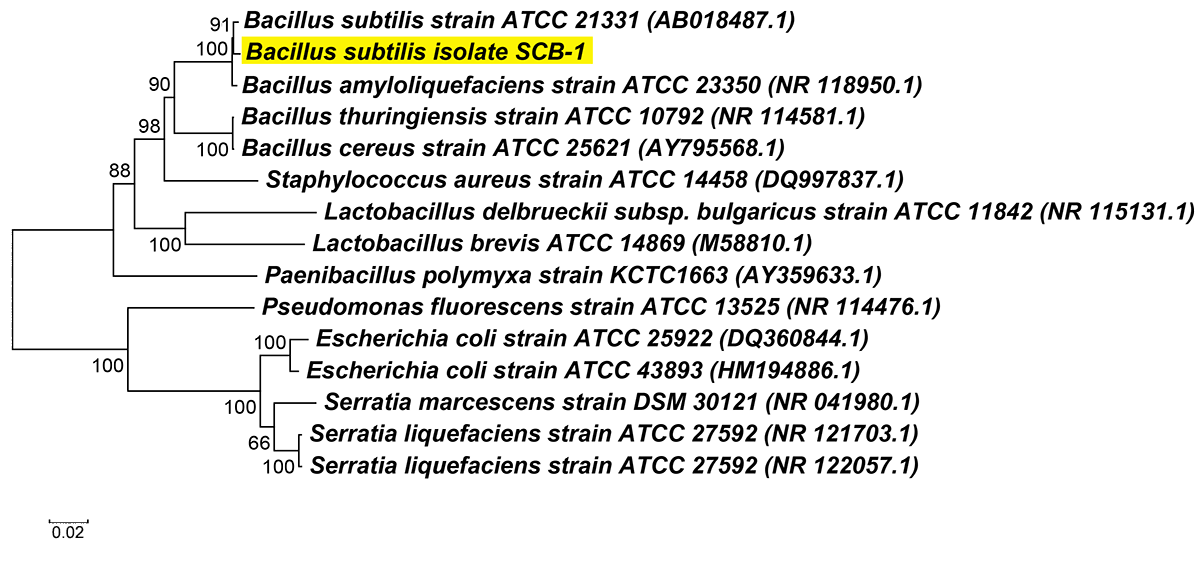


**Figure S1:** Phylogenetic relationship of *Bacillus subtilis* SCB-1 with their related bacterial strains based on the 16S rRNA gene sequence. The tree was constructed using Neighbour-Joining method incorporated in MEGA6 with 1000 bootstrap replications.


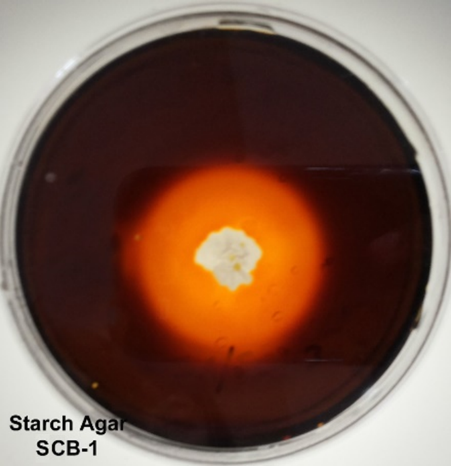

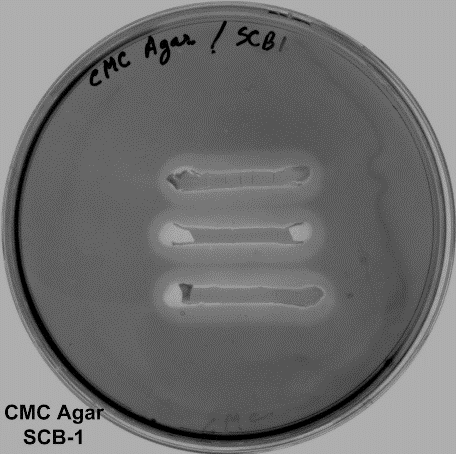

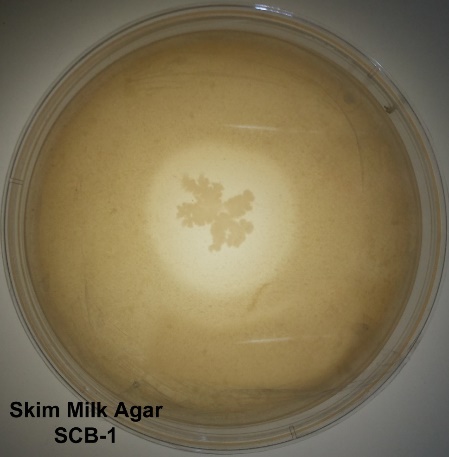


**Figure S2:** Hydrolytic enzyme activity of SCB-1 on solid medium. **A.** Amylase activity on starch agar medium. **B.** Cellulase activity on CMC agar medium. **C.** Protease activity on skimmed milk agar medium.


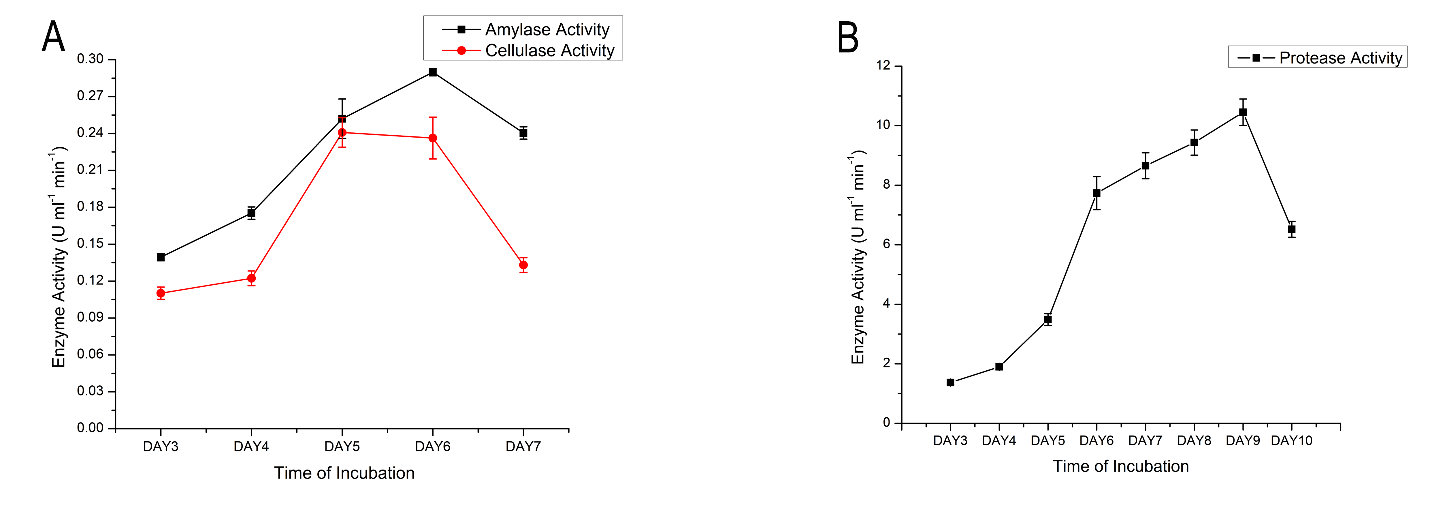

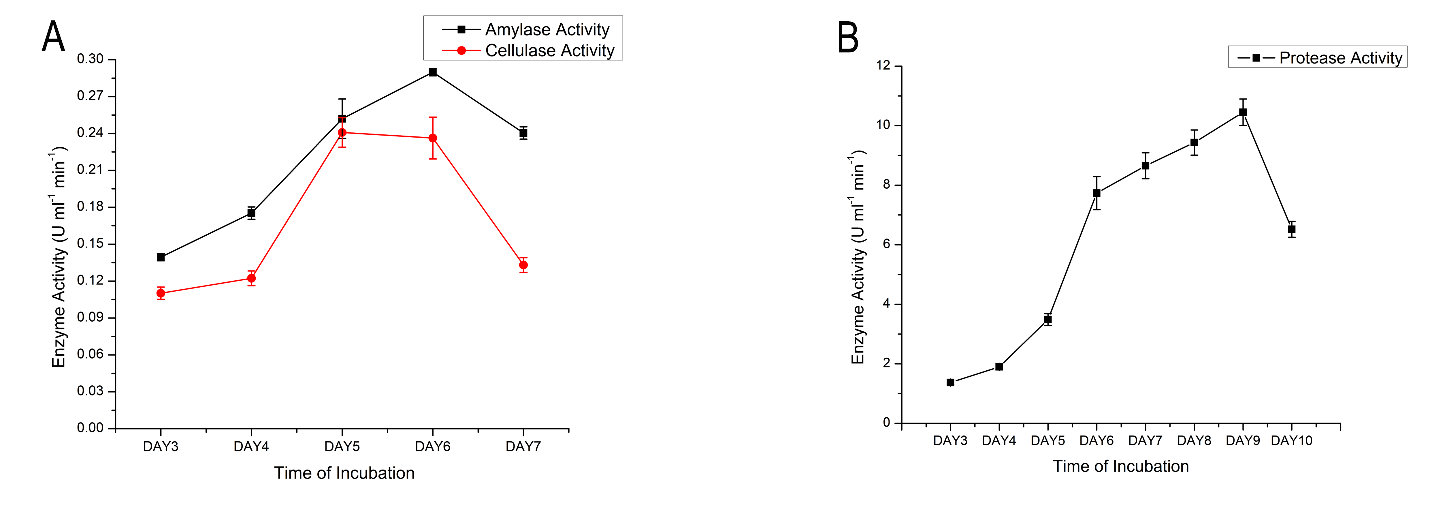


**Figure S3:** Enzyme activity against the incubation time of *Bacillus subtilis* SCB-1 in respective culture medium. Error bars represent the standard deviation from three replicates.
